# Optimal age of surgery for children with thyroglossal duct cysts: A single-institution retrospective study of 340 patients

**DOI:** 10.3389/fped.2022.1038767

**Published:** 2023-01-26

**Authors:** Yingli Wang, Gang Yang

**Affiliations:** ^1^ West China School of Nursing/Department of Hematology, West China Hospital, Sichuan University, Chengdu, China; ^2^ Department of Pediatric Surgery, West China Hospital, Sichuan University, Chengdu, China

**Keywords:** thyroglossal duct cyst, children, age, Sistrunk procedure, surgery

## Abstract

**Objective:**

The recommended age of surgery for thyroglossal duct cysts (TGDCs) in children is inconclusive. This study aimed to explore the optimal age of surgery by analyzing the natural history of the disease and the association between the age of surgery and postoperative complications.

**Methods:**

All TGDC patients who underwent a modified Sistrunk procedure at our hospital between March 2010 and May 2022 were reviewed retrospectively. The evaluation focused on the age of preoperative symptomatic cystic infection, pathological inflammation, postoperative wound infection, and recurrence.

**Results:**

Of the 340 patients included in the study, the median age of surgery was 47.5 months (IQR, 24.1–61.6). Preoperative symptomatic cystic infection and pathological inflammation frequencies were 27.1% (*n* = 92) and 48.5% (*n* = 165), respectively. The cumulative hazard of symptomatic cystic infection and pathological inflammation increased steadily with age. The ages of 50% cumulative incidence of symptomatic cystic infection and pathological inflammation were 97 months and 71 months, respectively. Postoperative wound infection was higher in patients of younger age (OR = 0.96, 95% CI, 0.93–0.98, *P* < 0.001) and with symptomatic cystic infection (OR = 8.16, 95% CI, 2.54–36.86, *P* = 0.002). There was no significant association between the age of surgery and recurrence.

**Conclusion:**

Although wound infection was weakly associated with younger age, the symptomatic cystic infection increasing with age has a more remarkable impact on wound infection after the Sistrunk procedure. The recurrence rate did not increase in young patients receiving surgery. Therefore, the Sistrunk procedure was safe and effective at a young age, and prompt operation in children with TGDC once diagnosed was reasonable.

## Introduction

Thyroglossal duct cyst (TGDC) is the most frequently encountered congenital abnormality of the neck in children (1, 2). Surgical excision is indicated to prevent infection, sinus or fistula formation, cosmetic defects, and malignancy (3). Both classic and modified Sistrunk procedures have been generally accepted as the standard treatment for TGDC (4, 5). Nevertheless, postoperative complications (4.4%–20.9%) and recurrence (4.1%–10.7%) remain significant even when the Sistrunk procedure is adhered to strictly (6–9).

Although much literature has reported experiences of surgery for TGDC based on institutional or national databases, debates exist regarding the recommended age of surgery (10, 11). It was reported that the recurrence rates were 1/2 and 1/3 when the children were operated on under the ages of 1 year and 2 years old, respectively (12). However, recent studies suggested that age may not be a risk factor for adverse outcomes (6, 13). Another factor raising concerns about the early operation is anesthesia-induced neurotoxicity in the developing brain. Therefore, parents and providers could postpone the procedures to avoid general anesthesia (14). Nevertheless, delayed treatment exposed the children to a higher risk of infection and postoperative complications. This study sought to assess the optimal age of surgery in children with TGDC by reviewing data in our center, with particular emphasis on the natural history of disease and the association between age and adverse outcomes following the Sistrunk procedure.

## Materials and methods

### Study population

A retrospective chart review was performed to evaluate patients with a diagnosis of TGDC in the Department of Pediatric Surgery, West China Hospital, Sichuan University, from March 2010 to May 2022. Inclusion criteria were age 15 years or younger and the performance of a primary modified Sistrunk procedure for suspected TGDC, with the final pathologic diagnosis of TGDC. The patients with redo Sistrunk surgery for recurrent TGDC were excluded. Other exclusion criteria included age greater than 15 years and any concurrent or alternative procedures such as isolated cyst excision, marsupialization, or incision with drainage performed. The Institutional Review Board of our hospital approved the study (ethical approval number, 2021-1902). The requirement for informed consent was waived because this study was based on retrospective chart reviews. Reporting followed guidelines from Strengthening the Reporting of Observational Studies in Epidemiology (STROBE).

### Clinical procedure

Standard preoperative evaluations included ultrasound examination for neck mass and thyroid. In all patients, the modified Sistrunk procedure was followed. It is defined as the excision of the cyst with the central portion of the hyoid bone and removal of a core of tissue in the suprahyoid region but with preservation of oral mucosa without entry into the oropharynx. The drain was not placed routinely.

### Data collection

Data including age, gender, symptoms, history of preoperative cystic infection, prior incision and drainage, procedure date, postoperative complications, recurrence, and histopathological findings were collected. Primary procedure was defined as the procedure performed in a patient with TGDC without a history of any prior surgical procedure attempting to remove it. Recurrence was defined as a recurring midline cyst or fistula requiring surgical excision. Pathologic inflammation was defined as the infiltration of inflammatory cells or granulomatous inflammation.

### Statistics analyses

The categorical variables were recorded as frequency counts and percentages. Continuous variables were recorded as means, standard deviations, medians, interquartile ranges (IQRs), and ranges. The Kaplan–Meier product-limit method was used to estimate the event or event-free rates for time-to-event endpoints, including the age of symptomatic infection and pathological inflammation. In our analyses, we considered the age of patients with pathological inflammation as a proxy for the time of asymptomatic infection. Differences between the groups were assessed using the Mann–Whitney *U* test for continuous variables and Pearson's *χ*^2^ test or Fisher's exact tests for categorical variables. Multivariable logistic regression analysis was performed to model the probability of an adverse event. Odds ratios and 95% confidence intervals (CIs) were reported. A significant difference was considered when the *P*-value was less than 0.05. All statistical analyses were performed with SPSS software (Statistical Package for the Social Sciences; IBM Corp., Armonk, NY) and R statistical software, R version 4.1.3, 2022 (R Foundation for Statistical Computing).

## Results

### Demographics

**Figure 1:**
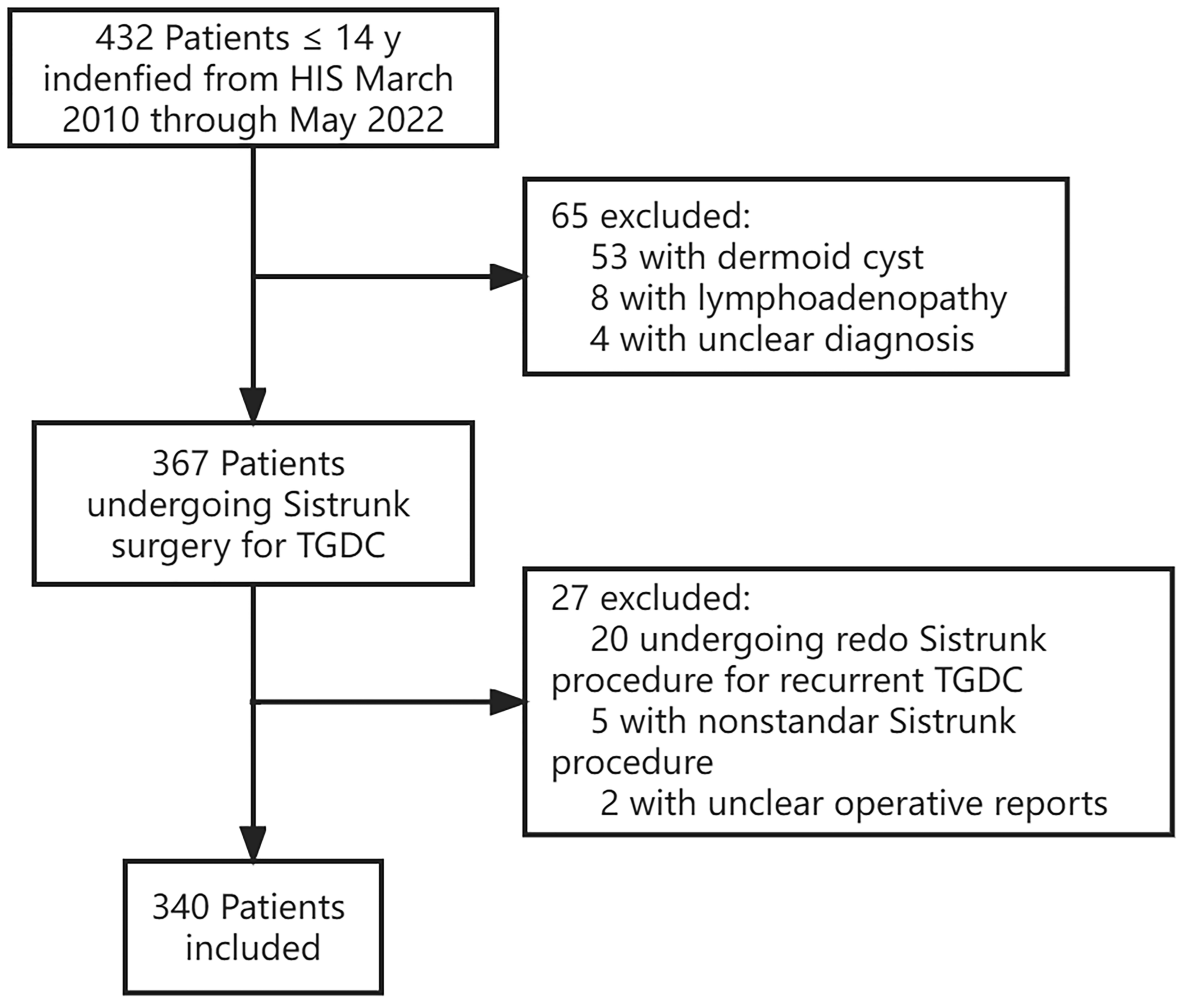
STROBE flow chart for selection of patients in the study. HIS, hospital information system; TGDC, thyroglossal duct cyst.

**Figure 2:**
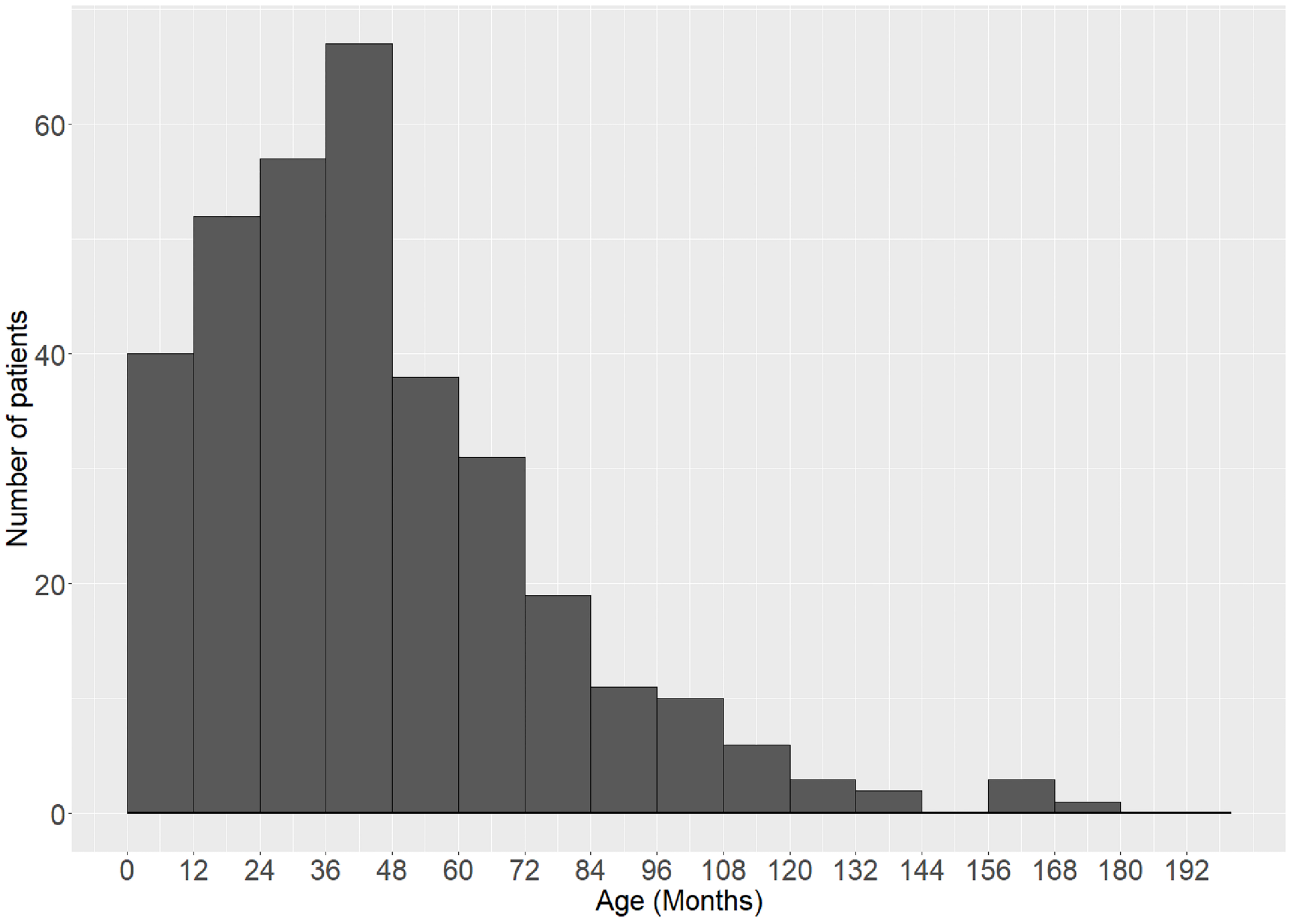
Distribution of included patients’ age at operation.

In total, 432 children were identified from the hospital information system. After excluding the redo Sistrunk procedure, nonstandard modified Sistrunk procedure, and pathological diagnosis other than TGDC, 340 children were included in the analysis (Figure 1). In the final cohort, the median age of presentation was 30.0 months (IQR 14.3–50.6). The operation was performed at 47.5 months (IQR 24.1–61.0) with 45.0% girls. The distribution of the age is shown in Figure 2. The time elapsed between the presentation and the procedure was 11.3 ± 14.3 months. The follow-up period ranged from 2 weeks to 5 years (median 6 months, IQR 4.1–15.0). The detailed demographic and clinical characteristics are given in Table 1.

**Table 1:** Demographics and clinical characteristics of patients.

Characteristics	
**Sex (*n*, %)**	
Male	187 (55.5%)
Female	153 (45.0%)
Age at operation (months) (median, IQR)	47.5 (24.1–61.0)
Age at presentation (months) (median, IQR)	30.0 (14.3–50.6)
Age and symptomatic infection (months) (median, IQR)	35.5 (14.3–57.4)
Presentation of infection (*n*, %)^a^	92 (27.1%)
Painful swelling	79/92 (85.9%)
Erythema/redness	72/92 (78.3%)
Fever	7/92 (7.7%)
Discharging sinus	31/92 (33.7%)
Age with pathological inflammation (months)	47.0 (24.0–60.0)
Mean operation time (min)	41.2 ± 13.9
Postoperative wound infection (*n*, %)	27 (7.9%)
Recurrence (*n*, %)	12 (3.5%)

^a^ Patient may have more than one presentation.

### Preoperative cyst infection and pathological inflammation

The median age for the notice of cysts by parents was 30.0 months (IQR, 14.1–50.9). Ninety-two patients (27.1%) had a preoperative symptomatic cyst infection history. The median age of cyst infection was 35.5 months (IQR, 14.3–57.4). The symptoms of infection included painful swelling (79/92, 85.9%), erythema/redness (72/92, 78.3%), fever (7/92, 7.7%), and discharging sinus (31/92, 33.7%). Eighteen patients (19.6%) were treated with incision and drainage (I&D) before the operation. Examination of the pathologic specimen showed signs of inflammation in 165 patients (48.5%), including inflammatory cell infiltration, chronic suppurative infection, and granuloma formation. The median age of the patient with pathological inflammation was 47.0 months (IQR, 24.0–60.0).

**Figure 3:**
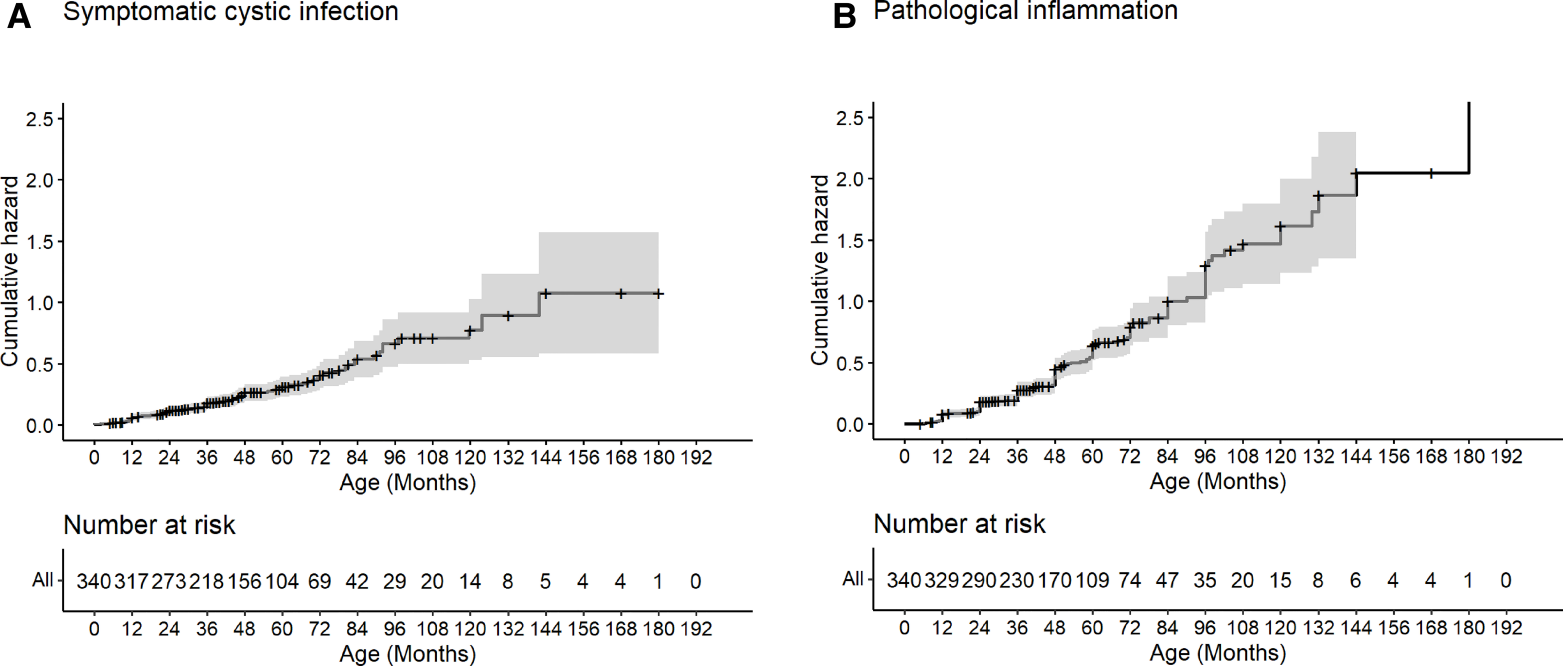
Cumulative hazard of symptomatic cystic infection and pathological inflammation. Kaplan–Meier analysis for the cumulative hazard of symptomatic cystic infection (**A**) and cumulative incidence of pathological inflammation (**B**) with age.

The cumulative hazard of symptomatic cystic infection and cumulative incidence of pathological inflammation in the overall cohort increased steadily with age (Figure 3). The ages of 50% cumulative incidence of symptomatic cystic infection and pathological inflammation was 97 months and 71 months, respectively.

### Postoperative complications

The mean operation time was longer in patients with a history of cyst infection (52.9 ± 18.3 vs. 38.3 ± 11.4 min, *P* < 0.001) and histological inflammation (44.7 ± 16.3 vs. 40.5 ± 13.9, *P* = 0.036). The overall postoperative complication rate following a Sistrunk procedure was 8.3%. No mortality, reintubation, or postoperative bleeding were identified. The most common complication was wound infection (27/340, 7.9%). There was one postoperative hoarseness of voice (1/340, 0.3%). Patients with wound infection were younger than those without wound infection at the age of surgery [27.1 months (IQR, 13.2–51.0) and 43.0 months (IQR, 24.2–63.8), *P* = 0.012]. Also, the occurrence of wound infection was higher in patients with preoperative cyst infection (77.8% vs. 22.7%, *P* < 0.001) or histological inflammation (88.9% vs. 45.0%, *P* < 0.001) (Table 2). Prior incision and drainage had no significant effect on wound infection rates (26.1% vs. 17.4%, *P* = 0.559). The Multivariable logistic regression found that younger age (OR = 0.96, 95% CI, 0.93–0.98, *P* < 0.001) and symptomatic cystic infection (OR = 8.16, 95% CI, 2.54–36.86, *P* = 0.002) were associated with the increasing risk of wound infection (Table 3).

**Table 2:** Univariable analysis of postoperative wound infection and recurrence of TGDC.

	Wound infection			Recurrence		
	Yes (*n* = 27)	No (*n* = 313)	*P*-value	Yes (*n* = 12)	No (*n* = 328)	*P*-value
Median age of surgery (IQR) (months)	27.2 (13.0–51.4)	43 (24–64)	0.012	27.9 (18.1–60.7)	42.0 (23.2–63.2)	0.328
Gender (male/female)	17/10	171/142	0.538	7/5	180/148	1.000
Interval time (months)^a^	9.4 ± 10.4	11.6 ± 14.6	0.527	9.5 ± 14.2	11.4 ± 14.4	0.650
Symptomatic infection	21 (77.8%)	71 (22.7%)	<0.001	4 (33.3%)	86 (26.2%)	0.525
Pathological inflammatory	24 (88.9%)	141 (45.0%)	<0.001	9 (75.0%)	156 (47.6%)	0.062

^a^ The duration from diagnosis to surgery.

**Table 3:** Multivariable logistic regression estimates of postoperative infection by age, sex, symptomatic infection, and pathological inflammation.

	Postoperative infection		Recurrence	
	OR (95%CI)	*P*-value	OR (95%CI)	*P*-value
Age (months)	0.96 (0.93–0.98)	<0.001	0.99 (0.97–1.01)	0.463
**Sex**				
Female	Ref.		Ref.	
Male	1.48 (0.59–3.89)	0.414	1.01 (0.31–3.53)	0.982
**Symptomatic infection**				
No	Ref.		Ref.	
Yes	8.16 (2.54–36.86)	0.002	0.64 (0.15–2.51)	0.517
**Pathological inflammation**				
No	Ref.		Ref.	
Yes	2.01 (0.36–11.31)	0.409	4.04 (0.96–20.23)	0.062

### Recurrence

Of 340 patients who underwent the primary procedure in our hospital, 12 developed recurrence (3.5%). The age, sex, symptomatic infection, and histological inflammation did not associate with the recurrence of TGDC (*P* > 0.05) (Tables 2, 3).

## Discussion

When determining the optimal age of surgery for TGDC, factors such as the natural disease course of TGDC and the safety and effectiveness of the surgery should be considered. The results of existing research about the potential risk factors contributing to the adverse events were mixed, and the recommended age of the Sistrunk procedure for pediatric TGDC is inconclusive. In this study, we used the survival analysis method to estimate the natural history of TGDC and the cumulative hazard for the occurrence of infection. We also analyzed the association of age with postoperative complications.

In our study, the peak age of presentation was younger than what was previously reported that TGDC presented in preschool-aged more commonly (15, 16). Typically, a bimodal distribution was observed, with the most common average age in children to 6 years and adults to 41–45 years (17). The age difference may be due to the following reasons. First, most patients' neck mass of TGDC was asymptomatic at presentation. The parents were difficulty recalling the accurate time of appearance. Second, most studies reported the age of diagnosis or procedure. The age of presentation should certainly be younger than that. As a congenital anomaly, thyroglossal duct remnants are observed in 7% during autopsy (17, 18). Cyst formation may be due to the inflammation stimulation of adjacent lymphoid tissue or obstruction of the duct (18). This may explain the high frequency of thyroglossal duct cysts in infants and preschoolers with the most prominent lymphoid tissue hyperplasia. Children are more liable to get all sorts of infectious diseases. Hence, resection of the cyst promptly is reasonable in children to reduce the risk of infection spreading from the oropharyngeal area.

The resection of the TGDC is to prevent complications associated with cystic infection. Preoperative infection reports vary from 17% to 46.2% (19, 20). The usual route of infection is via the mouth. It was speculated that older children are more likely to become infected. However, we could not identify any study analyzing cystic infection's age characteristics. In our series, the cumulative incidence and hazard of symptomatic cystic infection increased steadily with aging. Although antibiotics could treat most infections, some patients in our cohort progress to sinus formation by spontaneous perforation or I&D. The inflammation of the cyst can destroy the epithelial lining, stimulate the proliferation of granulation tissue, and disrupt the cyst wall (21). Although we did not find that preoperative infection and pathological inflammation would increase the recurrence after the Sistrunk procedure, the infection was positively correlated with postoperative wound infection. The repeated infection would make dissecting the cyst and hyoid challenging because of the fibrotic adhesions and result in unintentional airway injury (22). So, an early operation may prevent infection and lower the risk of the Sistrunk procedure in children.

In our study, wound infection was the most common postoperative complication. The rate of wound infection in our series (7.9%) was slightly higher compared with that recently reported (2.6%–4.1%) (6, 7, 23). The younger age was associated with a more increased risk of wound infection, but the effect size was mild. More importantly, the patients with symptomatic cystic infection had a much higher risk of wound infection.

Our study found that age was not a risk factor for the recurrence of TGDC after the Sistrunk procedure. In early studies, younger patients had a higher recurrence rate after surgery (12). However, more and more studies have verified that the recurrence rate was similar in any age group (13, 24). The high recurrence rate in early studies may be due to noncompliance with the Sistrunk procedure. Identifying the important anatomical markers in young patients was difficult because the ossified hyoid bone may override the top of the thyroid cartilage. For fear of unintentional injury, the midpart of the hyoid bone or the suprahyoid tissues may not be completely resected. As long as the anatomical relationship of the cyst and the anterior cervical landmarks been identified preoperatively, the Sistrunk procedure in young patients was safe and effective.

The parents feel concerned about general anesthesia in young patients. In our cohort, the operative time of the vast majority of patients was shorter than 1 h. It has been confirmed that there was no altered neurodevelopmental outcome at age 5 years in infants receiving less than 1 h of general anesthesia (25). However, very little is known about the impact of a long time or repeated exposures on subsequent behavior and cognitive function (26, 27). In 2016, the FDA warned that general anesthesia and sedation drugs used in children under 3 years of age or in pregnant women in their third trimester who were undergoing anesthesia for more than 3 h or repeated use of anesthetics might affect the development of children's brains (28). Our study also revealed that the operation time was longer in patients with cyst infection and histological inflammation. Though we did not evaluate the postoperative anesthesia outcome, the early operation may reduce the risk of cystic infection and shorten the anesthesia exposure time.

The observational and retrospective nature of this study is a significant limitation. Younger patients were more likely referred to an academic institution, and the choice of operation time was based on the discretion of surgeons and parents. These carried the potential selection bias. Secondly, the follow-up time may not be long enough in our study. It was reported that most recurrences occurred in the first postoperative year (29). The recurrence rate may be underestimated.

## Conclusion

Although postoperative wound infection was weakly associated with younger age, the symptomatic cystic infection increasing with age has a more remarkable impact on wound infection after the Sistrunk procedure. The recurrence rate did not increase in young patients receiving surgery. Therefore, the Sistrunk procedure was safe and effective at a young age, and prompt operation in children with TGDC once diagnosed was reasonable.

## Data Availability

The raw data supporting the conclusions of this article will be made available by the authors without undue reservation.
